# Validation of a microRNA profile in urine liquid biopsy with diagnostic and stratification value for bladder cancer classification, available through the open app BladdermiRaCan

**DOI:** 10.1186/s40164-025-00649-0

**Published:** 2025-04-11

**Authors:** Julia Oto, Raquel Herranz, Emma Plana, Javier Pérez-Ardavín, David Hervás, Fernando Cana, Patricia Verger, David Ramos-Soler, Manuel Martínez-Sarmiento, César D. Vera-Donoso, Pilar Medina

**Affiliations:** 1 Haemostasis, Thrombosis, Arteriosclerosis and Vascular Biology Research Group, Health Research Institute Hospital La Fe, Valencia, Spain; 2 Angiology and Vascular Surgery Service, La Fe University and Polytechnic Hospital, Valencia, Spain; 3 Department of Urology, La Fe University and Polytechnic Hospital, Valencia, Spain; 4 Biostatistics Unit, Health Research Institute Hospital La Fe, Valencia, Spain; 5 Department of Applied Statistics and Operations Research, and Quality, Universitat Politècnica de València, Valencia, Spain; 6 Department of Pathology, La Fe University and Polytechnic Hospital, Valencia, Spain; 7 School of Medicine, Universidad Católica de Valencia, Valencia, Spain

**Keywords:** Biomarker, BladdermiRaCan, Bladder cancer, Diagnosis, miR-eCLIP Immunoprecipitation, Liquid biopsy, microRNA, Prognosis, Stratification, Urine

## Abstract

We aimed to identify a profile of urine microRNAs (miRNAs) with diagnostic and stratification potential in the whole range of bladder cancer (BC) categories, to avoid current invasive, harmful and expensive procedures. We collected a first morning urine sample from the screening (35 BC patients and 15 age- and gender-matched controls) and validation cohorts (172 BC and 94 controls). In the screening stage we analyzed the expression level of 179 miRNAs by real-time reverse transcription quantitative PCR in urine supernatants. miRNA levels in each sample were normalized by the levels of the previously identified and stably expressed miR-29c-3p. We performed an ordinal regression for each miRNA with False Discovery Rate (FDR) adjustment to identify dysregulated miRNAs, and an ordinal elastic net logistic regression model to identify a miRNA profile for BC diagnosis and stratification with the software R (v3.5.1). Next, we validated the most dysregulated miRNAs, and empirically identified the real miRNA targets in BC cells by miR-eCLIP immunoprecipitation and sequencing. We identified 70 dysregulated miRNAs in BC patients (*p* < 0.05 FDR-adjusted). With the expression level of 7 miRNAs in urine (miR-221-3p, miR-93-5p, miR-362-3p, miR-191-5p, miR-200c-3p, miR-192-5p, miR-21-5p) we could stratify BC patients and control subjects. To enable the global use of our model, we developed the free BladdermiRaCan online tool. Furthermore, we identified miR-21-5p, miR-425-5p and miR-99a-5p as follow-up markers for BC relapse, and miR-21-5p and miR-221-3p as markers for metastasis. These miRNAs were also dysregulated in BC tissue sections from a subgroup of patients from which urine samples were studied. In conclusion, we have validated and patented a 7-miRNAs urine profile able to diagnose and stratify BC patients; BladdermiRaCan will enable the global use of our model. The experimentally verified target proteins identified for these miRNAs may unravel novel therapeutic targets.

**Supplementary Information:**

The online version contains supplementary material available at 10.1186/s40164-025-00649-0.

**To the editor**.

Bladder cancer (BC) represents 3% of all malignant tumors in adults worldwide and stands as the most lethal urological malignancy [1]. It also stands as one of the most costly cancers to monitor and treat, with muscle-invasive BC (MIBC) costing up to $150,000 per patient [2].

Presently, bladder ultrasound, cytology and cystoscopy are the gold standards for BC diagnosis and monitoring despite its low sensitivity or high invasiveness of the latest, respectively [3]. Consequently, novel urinary markers are being ascertained in liquid biopsies, such as microRNAs (miRNAs) [4] or cell-free DNA [5]. miRNAs play a role in BC development and may serve as biomarkers, though studies have mostly focused on individual stages like MIBC [6] or non-MIBC (NMIBC) [7]. We aimed to identify and validate a urine miRNA profile to diagnose and stratify BC, assess miRNAs linked to recurrence, metastasis, and cell subtypes, confirm their dysregulation in BC tissue, create a web tool for global use, and identify their target proteins via in silico and experimental methods.

We prospectively recruited 207 BC patients and 109 age- and sex-matched controls. Clinical characteristics are detailed in Table S1. No macroscopic evidence of hematuria or lipuria was evidenced in the samples collected for the study. To discard a potential contamination of the urinary miRNA content by erythrocytes, we assessed the hemolysis of the RNA samples with the level of miR-451a and miR-23a-3p (∆C_t_ (miR-23a-3p – miR-451a), where values > 7 indicate hemolysis) (*miRNAs as BC biomarkers: Screening stage*, Supplementary Material). Only 3 samples of low-grade tumors showed a limited degree of hemolysis: 2 TaG1 patients with values of 8.24 and 10.33, respectively, and a T1G3 patient with a value of 7.28. In the screening stage, 157 miRNAs were successfully quantified in the urine supernatant of 35 BC patients and 15 controls. Univariate ordinal regression identified 70 dysregulated miRNAs (*p* < 0.05 FDR-adjusted) (Table S2, Figure S1). The most significantly dysregulated miRNAs with FDR adjustment were miR-30a-5p, miR-425-5p, miR-99a-5p, miR-23a-3p, miR-215-5p and miR-10b-5p.

Next, we adjusted an ordinal elastic net logistic regression model for BC diagnosis and staging including as variables the expression of all 179 miRNAs comprised in the panel. This model incorporates a penalty term at the model-fitting step that helps to improve the model’s accuracy and generalization capacity by selecting the most relevant predictors and handling multicollinearity. The model selected 7 miRNAs as predictors (miR-93-5p, miR-362-3p, miR-191-5p, miR-200c-3p, miR-192-5p, miR-21-5p and miR-221-3p), and predictions are made considering the levels of the 7 miRNAs collectively with the detailed formula and applying a specific weight to each variable (Fig. 1A-C).

In the validation stage, we quantified the expression of the 6 main dysregulated miRNAs and the 7 predictor miRNAs in an independent cohort of 172 BC patients and 94 controls, including G2 tumors to cover the full BC spectrum. Expression differences were validated using univariate ordinal regression models. Our predictive model accurately diagnoses and stages BC, with strong agreement between predicted and observed values (Fig. 1D-F).

These results were patented under the name “microRNA profile in urine for bladder cancer diagnosis and staging” (patent number 202230663). Our predictive model is readily accessible through the open-access BladdermiRaCan application hosted at https://remote.iislafe.san.gva.es/sample-apps/bladder_miracan/ (Figure S2).

**Fig. 1:**
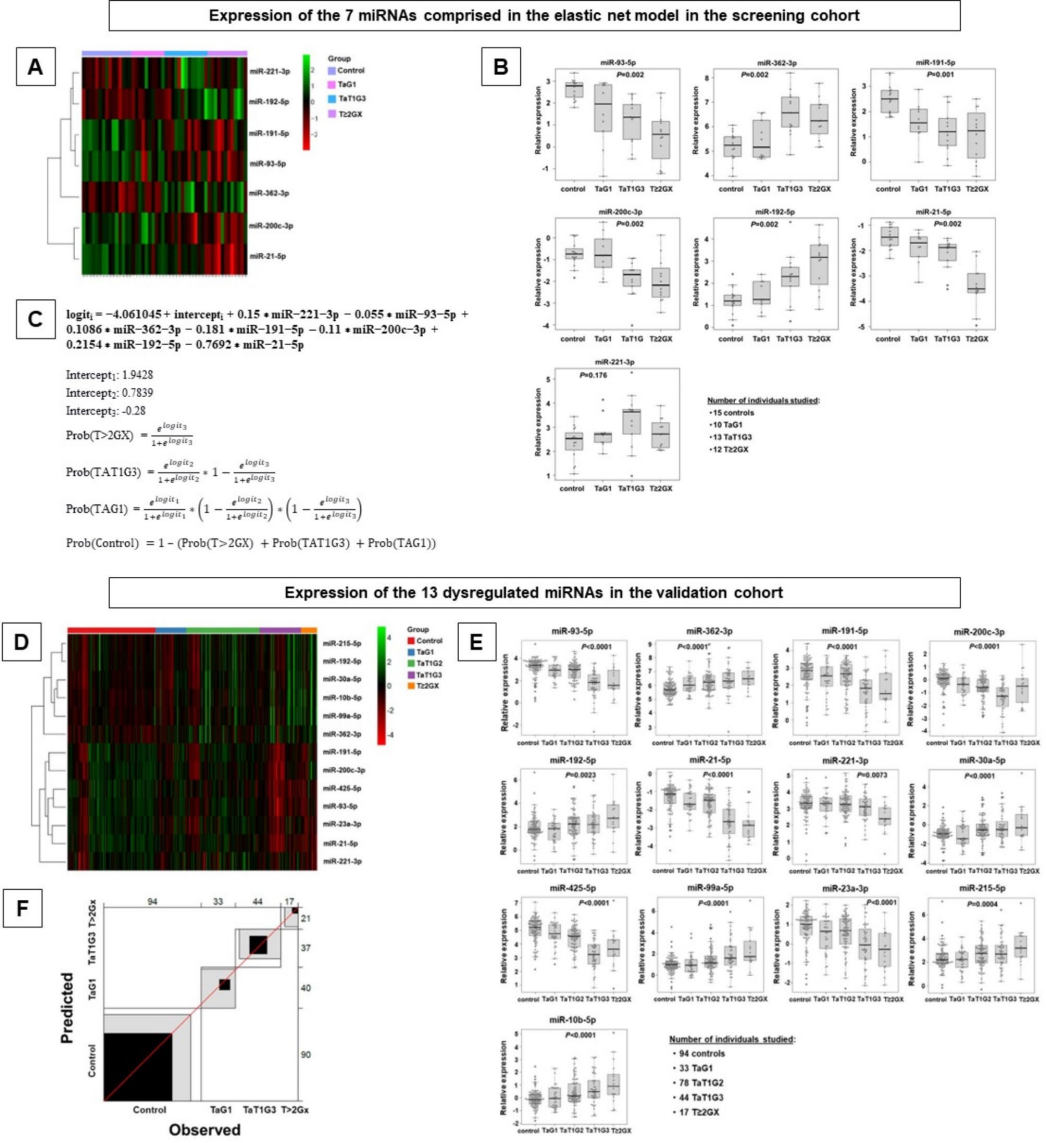
Expression level of the dysregulated miRNAs in the screening and validation cohorts. Expression of the 7 miRNAs comprised in the elastic net logistic regression model for BC diagnosis and staging. **A** Heatmap, green represents relative miRNA underexpression and red represents relative overexpression. **B** Relative miRNA expression in the different BC categories and controls. Due to the normalization strategy employed, a lower relative expression value indicates a higher expression of the miRNA of interest (see Statistical analysis, Supplementary Material). Global FDR-adjusted *p*-values of the ordinal regression model are depicted for each miRNA, and pairwise differences among groups are detailed in Table S3. **C** The formula to calculate the probability of belonging to a given category (T ≥ 2GX, TaT1G3, TaG1 or control). Expression of the 6 main dysregulated miRNAs and the 7 predictor miRNAs in the validation cohort. **D** Heatmap, green represents relative miRNA underexpression and red represents relative overexpression. **E** Relative expression in the different BC categories and controls (follow the interpretation detailed for panel B). Global FDR-adjusted *p*-values of the ordinal regression model are depicted for each miRNA, and pairwise differences among groups are detailed in Table S4. **F** Bangdiwala agreement plot that represents the agreement between predicted and observed values obtained in the validation cohort with our ordinal regression model. Perfect matches between predicted and observed values are resented as black squares, adjacent categories are represented as grey squares and lack of match are represented as white squares. Rho value of the validated model = 0.61, Bangdiwala weighted value = 0.62, Bangdiwala unweighted value = 0.42, reflecting a high coincidence between predicted and observed values for each individual studied. When selecting the most probable category with the model and considering only controls vs. any BC group, sensitivity and specificity were 0.75 and 0.70, respectively

Next, Next, we quantified in tissue all 13 dysregulated miRNAs, with results matching the trends seen in urine. The following miRNAs were significantly dysregulated in tissue: miR-93-5p, miR-21-5p, miR-30a-5p and miR-10b-5p (Figure S3).

Additionally, we observed a dysregulation of miR-21-5p, miR-425-5p and miR-99-5p in the sample collected right before relapse diagnosis, compared to the last sample collected from patients with tumor remission and controls (Figure S4A). Remarkably, in patients with remission, the expression of these miRNAs at the end of follow-up normalized to the levels of controls. We also found a higher expression of miR-21-5p and miR-221-3p in BC patients with metastasis (Figure S4B). In fact, their expression increased with the degree of BC severity in the validation cohort. Finally, we evidenced a significant increase in miR-93-5p, miR-425-5p, miR-21-5p and miR-200c-3p in BC patients with in situ BC (Figure S4C) compared to patients with papillary BC.

Finally, we analyzed the targets of the dysregulated miRNAs with two approaches: in silico and empirically. According to the in silico target identification, all the studied miRNAs have validated targets related to BC (Table S5). The empirical miR-eCLIP analysis conducted in T24 BC cells rendered a total of 26,622,109 chimeric reads in which 270 miRNAs are bound to 1,071 different mRNA targets in 6,941 genetic locations. Due to space limitations, the main 4 targets of our validated miRNAs are detailed in Table 1.

**Table 1:** Main targets of the 13 dysregulated miRNAs in BC identified by miR-eCLIP analysis. The 4 target genes with the highest number of chimeric reads per miRNAs are detailed. miR-eCLIP quantitatively detects functional miRNAs target genes, higher miRNAs-mRNA chimeric read coverage indicates increased binding strength and increased repression in corresponding RNA-seq experiments

miRNA	**Total number of target genes**	Total chimeric reads	Main 4 target genes			
			Target gene	Number of chimeric reads	Binding site in mRNA	Gene previously related to BC
miR-221-3p	114	629,780	*MIDN*	116,278	3' UTR	no
			*PODXL*	27,372	3' UTR	yes
			*NUCKS1*	26,150	3' UTR	no
			*CCN1*	22,743	3' UTR	no
miR-93-5p	73	194,289	*CCND1*	14,399	3' UTR	yes
			*TGFBR2*	12,891	3' UTR	yes
			*MIDN*	11,715	3' UTR	no
			*HMGA2*	11,327	3' UTR	yes
miR-362-3p	1	2,000	*HMGB2*	2,000	3´UTR	yes
miR-191-5p	18	29,203	*IFFO2*	5,314	3´UTR	no
			*HOXB2*	2,994	3´UTR	yes
			*MESD*	2,500	3´UTR	no
			*CXCL6*	2,000	3´UTR	yes
miR-200c-3p	0	0*	-	-	-	-
miR-192-5p	0	0*	-	-	-	-
miR-21-5p	182	918,909	*MT-TM*	298,085	Other	no
			*MALRD1*	109,070	Other	yes
			*ZFTA*	28,085	3' UTR	no
			*ZCCHC3*	27,181	3' UTR	no
miR-30a-5p	36	73,694	*GNAI2*	4,894	3' UTR	no
			*ADRB2*	4,109	3' UTR	yes
			*PLAGL2*	3,962	3' UTR	yes
			*CCDC71L*	2,885	3' UTR	no
miR-425-5p	9	51,088	*CCN1*	26,969	3' UTR	no
			*PPP2CB*	14,733	3' UTR	yes
			*ANXA2*	2,462	3' UTR	yes
			*STC2*	1,924	3' UTR	no
miR-99a-5p	3	5,000	*PPIA*	2,000	CDS	no
			*AGO2*	2,000	3´UTR	yes
			*MYLIP*	1,000	3´UTR	no
miR-23a-3p	175	1,552,337	*HMGB2*	173,960	3' UTR	yes
			*TUBB4B*	72,967	CDS	no
			*HAS2*	64,507	3' UTR	yes
			*MCFD2*	57,361	3' UTR	no
miR-215-5p	1	1,000	*MT-RNR2*	1,000	Other	yes
miR-10b-5p	7	15,707	*CMPK1*	8,831	3' UTR	no
			*GCLM*	2,551	3' UTR	yes
			*H3-3B*	1,325	3' UTR	no
			*RPS28P7*	1,000	Other	no

* miRNAs with a very low expression level in T24 BC cells are not present in AGO2, thus rendering 0 chimeric reads. miR-21-5p was the most abundant miRNA in the chimeric reads, miR-23a-3p the 4th, and miR-221-3p the 13th, highlighting their importance in BC regulation. Among the main targets, *MIDN*, *CCN1* and *HMGB2* are regulated by two miRNAs, suggesting a role in BC development

## Discussion

The need for reliable non-invasive biomarkers to diagnose and monitor BC is widely recognized to address the limitations of current techniques, including low sensitivity, high invasiveness, and substantial costs. We have created, validated in a large cohort, and patented a model comprising 7 urine miRNAs (miR-221-3p, miR-93-5p, miR-362-3p, miR-191-5p, miR-200c-3p, miR-192-5p and miR-21-5p) able to identify and stratify BC patients from healthy subjects. We identified other 63 FDR-adjusted dysregulated miRNAs, and validated the 6 most significant (miR-30a-5p, miR-425-5p, miR-99a-5p, miR-23a-3p, miR-215-5p and miR-10b-5p). Although previous studies have identified urine miRNAs as novel non-invasive biomarkers to improve BC diagnosis and monitoring [6–8], our study stands out by encompassing the full range of BC grades and stages. This comprehensive approach enables a more refined patient stratification beyond the conventional classification into low- or high-risk patients [9], or discriminating NMIBC from MIBC [6, 10, 11]. Particularly, ours is the first study that identified a miRNA profile able to effectively stratify BC patients. We also identified potential miRNA markers for BC relapse, metastasis, or specific subtypes; although larger validation studies should be performed to confirm our preliminary findings. Remarkably, several of these miRNAs have been previously associated to BC. miR-221 may have a diagnostic potential for BC in urine [12] and urine sediments [13]; and its maturation modulated by METTL3 leads to PTEN inhibition, BC growth and poor BC patient prognosis [14]. miR-200c-3p and miR-21-5p are dysregulated in urine [12], urine exosomes and paired BC tissue [15], being miR-200c-3p proposed as diagnostic and staging marker [16]. We proved that urine serves as liquid biopsy for BC as it reflects the miRNAs expression trend in the tumor microenvironment. However, nine miRNAs were not significantly dysregulated in tissue samples as they are in urine. This effect could be caused by a different rate of release into urine among miRNAs depending on their involvement in tumor-specific processes, and also by the reduced number of tissue specimens analyzed compared to urine samples, which represents a limitation for this comparison. Hematuria is a common sign of BC, particularly in high-grade tumors, what may affect the urine miRNA content and could have influenced the accuracy of our model. However, no macroscopic evidence of hematuria was evidenced in the samples collected for the study, thus the risk of erythrocyte contamination of the miRNA content in our study samples was minimum. Future studies should be conducted in urine samples with hematuria to confirm the diagnostic and stratification ability of our model in this scenario. In addition, future studies including patients with suspicion of BC as controls should be conducted, which represents a limitation of our control group.

We evidenced that all the miRNAs proposed have validated targets related to BC development and progression, and ten of the thirteen miRNAs studied had predicted targets along the BC pathway. As computational predictions produce many false positives, we sequenced the unions miRNA-mRNA in T24 BC cells and 270 miRNAs regulate 1,071 proteins. Expectedly, with miR-eCLIP we could only validate 8% of the target predictions, highlighting the importance of experimental target identification. Remarkably, we identified many more dysregulated proteins in BC. Of the 13 miRNAs of interest, miR-21-5p, miR-23a-3p and miR-221-3p are top regulators in BC cells, and 11 of them had at least one main target previously related to BC, highlighting the relevance of these miRNAs in BC. Among the most regulated targets, CCND1 was the main target of miR-93-5p and miR-245-5p, regulation that was also computationally predicted. Herein we evidenced that is also regulated by miR-23a-5p. Future functional studies in BC cell lines are needed to unravel the role of these miRNAs in the development and progression of BC.

In summary, the main target of our model would be patients with clinical suspicion for BC. The validation process conducted herein supports its potential translation into clinical practice. We are currently validating its study with the diversity of technology available in a clinical laboratory setting. Compared to other urinary tests proposed that have not reached the clinical setting, our non-invasive, robust, and affordable model (~ 175€/sample) could significantly reduce healthcare expenditure and enhance BC management by lowering the need for invasive cystoscopies in early detection and monitoring. To support the global use in a clinical environment, we developed the open BladdermiRaCan app. The experimentally confirmed miRNA targets in BC cells using miR-eCLIP immunoprecipitation and sequencing, may open new venues for novel therapeutic targets for BC.

## Electronic supplementary material

Below is the link to the electronic supplementary material.

Supplementary Material 1

## Data Availability

All relevant data are provided within the article and its supplementary data files. Additional data like statistical code are not available to other researchers as are under patent number 202230663.

## Abbreviations

BC: Bladder cancer
FDR: False discovery rate
miRNA: microRNA
MIBC: Muscle-invasive bladder cancer
NMIBC: Non-muscle-invasive bladder cancer
