# Supplementary material for: Validation of a microRNA profile in urine liquid biopsy with diagnostic and stratification value for bladder cancer classification, available through the open app BladdermiRaCan
Source: Exp Hematol Oncol. 2025 Apr 11;14:58. doi: 10.1186/s40164-025-00649-0 (PMC11987439; doi:10.1186/s40164-025-00649-0)
Supplement: Supplementary file 1 — Supplementary Material 1 [file 40164_2025_649_MOESM1_ESM.docx]

**SUPPLEMENTARY MATERIAL**

**Validation of a microRNA profile in urine liquid biopsy with diagnostic and stratification value for bladder cancer classification, available through the open app BladdermiRaCan**

Julia Oto^1^, Raquel Herranz^1^, Emma Plana^1,2^, Javier Pérez-Ardavín^3^, David Hervás^4,5^, Fernando Cana^1^, Patricia Verger^1^, David Ramos-Soler^6^, Manuel Martínez-Sarmiento^3^, César D. Vera-Donoso^3,7^, Pilar Medina^1^.

^1^ Haemostasis, Thrombosis, Arteriosclerosis and Vascular Biology Research Group, Health Research Institute Hospital La Fe, Valencia, Spain.

^2^ Angiology and Vascular Surgery Service, La Fe University and Polytechnic Hospital, Valencia, Spain.

^3^ Department of Urology, La Fe University and Polytechnic Hospital, Valencia, Spain.

^4^ Biostatistics Unit. Health Research Institute Hospital La Fe, Valencia, Spain.

^5^ Department of Applied Statistics and Operations Research, and Quality. Universitat Politècnica de València. Valencia, Spain.

^6^ Department of Pathology, La Fe University and Polytechnic Hospital, Valencia, Spain.

^7^ School of Medicine, Universidad Católica de Valencia. Valencia, Spain.

**CORRESPONDENCE:**

Dr. Pilar Medina, PhD

IIS La Fe-Hospital Universitario y Politécnico La Fe

Torre A, 5ª Planta, Lab. 5-09

Av. Fernando Abril Martorell 106

46026 Valencia, Spain

Phone: 34-961246636

E-mail: [medina_pil@gva.es](mailto:medina_pil@gva.es)

**MATERIALS AND METHODS**

**Patients and control subjects**

Two-hundred and seven BC patients were recruited between April 2016 and January 2020 at La Fe University and Polytechnic Hospital (Valencia, Spain). One-hundred and nine age- and sex-matched volunteers were also recruited as controls, who were patients of the Urology Service who were being examined for non-malignant disorders (e.g. cystitis, benign prostate hyperplasia) which could be differential diagnosis of bladder cancer. These patients underwent an ultrasound scan to rule out the presence of urological malignancies or other alterations. Once the motive of consult was resolved, a first-morning urine sample was collected. Patients and controls were clinically followed-up until May 2021.

# Patients with a clinical suspicion of BC were enrolled in the study, either at first diagnosis or at recurrence. Urine samples from patients were collected right before any surgical approach (transurethral resection of bladder tumor or cystectomy) and none were under treatment for BC. Patients were excluded from the study in the absence of histological confirmation. In a small subset of 17 BC patients from the validation cohort, urine samples were collected every 6 months for 2 years to identify miRNAs related to relapse.

Pre-operative clinical staging was performed through physical examination, urine cytology and CT scans of the chest, abdomen and pelvis (in case of invasive BC). The tumor histological classification was done according to grade in the WHO 1973 and 2004 classifications. Demographic and clinical data were collected. The presence of metastasis was assessed using imaging and the cellular subtype using histopathology.

All participants provided written informed consent according to protocols approved by the ethics review board at La Fe University and Polytechnic Hospital (Ref. 2014/0314). The study was performed according to the declaration of Helsinki, as amended in Edinburgh in 2000.

**Urine collection**

# A first morning urine sample of approximately 25 to 50 ml was collected in sterile containers from all participants. The urine was kept at 4 ºC until processing. Urine was centrifuged at 805 x g for 5 min at 4 ºC to remove cellular debris, thus avoiding any cellular contribution to the miRNAs isolated from urine (e.g. from erythrocytes or leukocytes). Urine supernatant was aliquoted and frozen at -80 ºC until analyzed. The concentration of creatinine in urine was measured by colorimetric clinical laboratory standardized methods.

**RNA isolation**

Total RNA (including miRNAs) from 600 µl of urine was isolated from urine supernatants using the miRNeasy Mini Kit (Qiagen, Hilden, Germany) following manufacturer´s instructions with several modifications optimized by our group [1]. During the isolation, an RNA carrier (tRNA, Ambion, Bleiswijk, TheNetherlands) was included to enhance the yield, and a mixture of synthetic miRNAs (Spike-in kit UniRT, Qiagen) was included to control for RNA isolation efficiency, cDNA synthesis and inter-plate quantitative PCR (qPCR) performance.

Total RNA from four to ten 5 µm sections (depending on tumor size) of formalin-fixed paraffin-embedded (FFPE) BC tissue was isolated per patient with the miRNeasy FFPE kit (Qiagen) following manufacturer´s instructions. Tumor tissue areas and adjacent healthy tissue were carefully selected by a pathologist based on hematoxylin-eosin staining.

The concentration and purity of the RNA was assessed by spectrophotometric quantification with the NanoDrop ND-1000 (Thermo Fisher Scientific, Wilmington, DE, USA) by means of the ratios A260/A280 and A260/230. We considered as standards of RNA purity an A260/A280 value ~2.0 and an A260/230 value ~2.2, as different values can indicate the presence of contaminants like phenol, trizol, guanidine isothiocyanate, proteins, EDTA and carbohydrates, among others. In our samples, the A260/280 was 1.996 ±0.107 (mean ± standard deviation). RNA was stored at -80 ºC until used.

**Quantification of the expression level of miRNAs**

To identify BC biomarkers, the expression level of miRNAs was quantified by real-time reverse transcription qPCR (RT-qPCR) in a LightCycler 480 II (Roche, Mannheim, Germany) in two stages:

*miRNAs as BC biomarkers: Screening stage*

Based on the yield and quality of the isolated RNA, 35 BC patients and 15 controls were selected, and miRNAs expression level was studied. The Universal cDNA Synthesis Kit II (Qiagen) was used for retrotranscription. As no specific panels had been designed for urine samples, the expression level of 179 miRNAs frequently present in biofluids was quantified with the Serum/Plasma Focus microRNA PCR Panel V5 (Qiagen), with the ExiLENT SYBR Green Master Mix (Qiagen) as previously reported [2–5]. Furthermore, each panel includes the following internal controls: 5 synthetic RNAs of the RNA Spike-in-kit aimed to monitor the RNA isolation and cDNA synthesis, and an inter-plate calibrator in triplicate and a negative control to evaluate qPCR performance. The potential hemolysis of the sample can be assessed with the level of miR-451a and miR-23a-3p comprised in the panel. If ∆C_t_ (miR-23a-3p – miR-451a) is >7, it may be an indication of contamination of the sample.

To normalize the expression level of each miRNA studied in urine, we employed the expression of miR-29c-3p as we demonstrated it to be a robust normalizer for urine miRNA studies in BC [3] and no differences in expression occurred among clinical groups.

*miRNAs as BC biomarkers:Validation stage*

Once identified in urine the main dysregulated miRNAs and a miRNA profile potentially able to diagnose and stratify BC patients, their expression level was quantified in an independent and larger cohort of 172 BC patients and 94 controls at inclusion in duplicate. For that aim, specific miRNA miRCURY LNA miRNA PCR Assays (Exiqon) were used. Each miRNA was measured in duplicate and a standard deviation (SD) <0.5 was considered satisfactory.

*Dysregulation of miRNAs according to BC relapse, metastasis and cellular subtypes*

We also analyzed the variation of urine miRNA expression depending on BC relapse in 51 serial samples from 17 BC patients of whom 6 patients had BC recurrence, in the sample right before relapse diagnosis and in the last sample collected from BC patients without recurrence and from controls. In addition, we analyzed the expression of the dysregulated miRNAs according to the presence of metastasis (15 patients with metastasis *vs.* 157 patients without metastasis) and cellular subtype (14 urothelial-*in situ* *vs.* 158 urothelial-papillary).

*Analysis of the dysregulated miRNAs in FFPE BC tissue sections*

Furthermore, the expression of the dysregulated miRNAs was quantified in FFPE tissue sections from 30 BC patients of the screening stage from whom tumor tissue sections were available. In addition, adjacent healthy bladder urothelium was available from two of these patients. miRNA expression levels were quantified as aforementioned. The selection of the most stable normalizer among all samples was performed with the comprehensive tool RefFinder that comprehends the computational programs geNorm, Normfinder, BestKeeper and the comparative delta-Ct method (<https://www.ciidirsinaloa.com.mx/RefFinder-master/>) [6]. The candidate normalizers evaluated were those proposed by the manufacturer for tissue samples (miR-103a-3p, miR-423-5p and miR-423-3p) and miR-29c-3p, the urine normalizer.

**Shiny app development**

To enable any researcher worldwide to predict BC diagnosis and stage of their patients using our model, we created the open web page app BladdermiRaCan (<https://remote.iislafe.san.gva.es/sample-apps/bladder_miracan/>). The interface was created with Shiny (v1.4) implemented with a graphical user interface (GUI), thus it can be used by researchers without knowledge of the R language.

**Identification of miRNAs´ targets**

*In silico identification*

Once we selected a miRNA profile with diagnostic and staging potential, we identified their validated and predicted target proteins related to BC using the commonly used database miRWalk 2.0 (<http://mirwalk.umm.uni-heidelberg.de/>) that comprehends 12 miRNA-target prediction programs [7,8]. Next, these targets were integrated within the *bladder cancer* pathway from Kyoto Encyclopedia of Genes and Genomes (KEGG) (<https://www.genome.jp/kegg/>) to further establish the potential involvement of these miRNAs in BC.

*Empirical identification*

As computational predictions produce many false positives, in addition, we experimentally identified the real targets of these miRNAs in BC cells with the miR-eCLIP assay. This technique identifies direct miRNA target sites by sequencing miRNA-mRNA chimeras occurring in the cells, thus guaranteeing that these proteins are truly regulated by these miRNAs in these BC cells. The standard eCLIP protocol [9] was modified to enable chimeric ligation of miRNA and mRNA [10]. Studies were performed in duplicates by Eclipsebio (San Diego, <https://eclipsebio.com/>). While plated, approximately 20 x 106 T24 cells (ATCC) were UV crosslinked at 400 mJoules/cm^2^ with 254 nm radiation, pelleted, snap frozen on dry ice and stored until use at -80°C. Cell pellets were then lysed with 1 mL of eCLIP lysis mix and sonicated (QSonica Q800R2) for 5 minutes, 30 seconds on / 30 seconds off with an energy setting of 75% amplitude, followed by digestion with RNase-I (Ambion). A primary mouse monoclonal AGO2/EIF2C2 antibody (sc-53521, Santa Cruz Biotechnology) was incubated for 1 hour with magnetic beads pre-coupled to the secondary antibody (M-280 Sheep Anti-Mouse IgG Dynabeads, Thermo Fisher 11202D) and added to the homogenized lysate for overnight immunoprecipitated at 4°C. Following overnight immunoprecipitation, 2% of the sample was taken as the paired size-matched input with the remainder magnetically separated and washed with eCLIP high stringency wash buffers. Chimeric ligation was then performed on-bead at room temperature for 1 hour with T4 RNA ligase (NEB). Immunoprecipitated samples were then dephosphorylated with alkaline phosphatase (FastAP, Thermo Fisher) and T4 PNK (NEB) and an RNA adapter was ligated to the 3′ ends. Immunoprecipitated and input samples were cut from the membrane at the AGO2 protein band size to 75 kDa above. Western blot was visualized using anti-AGO2 primary antibody (50683-RP02, SinoBiological) at a 1:2000 dilution, with TrueBlot anti-rabbit secondary antibody (18-8816-31, Rockland) at 1:6000 dilution. RNA adapter ligation, immunoprecipitated -western, reverse transcription, DNA adapter ligation, and PCR amplification were performed as previously described. Sequencing was performed as SE122 on the NextSeq 2000 platform.

After sequencing, samples were processed with Eclipsebio's proprietary analysis pipeline (v1). UMIs were pruned from read sequences using umi_tools (v1.1.1). Next, 3' adapters were trimmed from reads using cutadapt (v3.2). Reads were then mapped to a custom database of repetitive elements and rRNA sequences. All non-repeat mapped reads were mapped to the genome (UCSC version GRCh38/hg38) using STAR (v2.7.7a). PCR duplicates were removed using umi_tools (v1.1.1). AGO2 eCLIP peaks were identified within eCLIP samples using the peak caller CLIPper (v2.0.1). For each peak, IP versus input fold enrichments and *p*-values were calculated. miRNAs from miRBase (v22.1) were "reverse mapped" to any reads that did not map to repetitive elements or the genome using bowtie (v1.2.3). The miRNA portion of each read was then trimmed, and the remainder of the read was mapped to the genome using STAR (v2.7.7a). PCR duplicates were resolved using umi_tools (v1.1.1), and miRNA target clusters were identified using CLIPper (v2.0.1). Each cluster was annotated with the names of miRNAs responsible for that target. Peaks were annotated using transcript information from GENCODE release 35 (GRCh38.p13) with the following priority hierarchy to define the final annotation of overlapping features: protein coding transcript (CDS, UTRs, intron), followed by non-coding transcripts (exon, intron).

**Statistical analysis**

Data were summarized using mean (standard deviation) and median (1^st^, 3^rd^ quartile) in the case of continuous variables and relative and absolute frequencies in the case of categorical variables. miRNA values were normalized by subtracting from the raw Ct values for each sample the corresponding Ct values of the normalizer miRNA: miR-29c-3p for urine samples and miR-103a-3p for FFPE tissue sections; accordingly, a lower relative expression value indicates a higher expression of the miRNA of interest. An elastic net penalized ordinal regression model was adjusted to the screening cohort for discriminating between the different stages and grades using as potential predictors the miRNAs from the panel. Additionally, univariate ordinal regression models were adjusted for each miRNA and *p*-values were adjusted for multiple comparisons using false discovery rate (FDR). Results from the screening cohort were validated in an independent cohort of patients. Our predictive model allows for the diagnosis of BC and staging of the patient across the whole range of BC categories (ordinal regression); therefore, its accuracy cannot be evaluated using ROC curves as it only allows the comparison of two groups. Accordingly, predictions were made using the ordinal regression model fitted on the screening cohort and the discriminative power of the model was assessed by estimating the rank correlation coefficient (rho value) between predicted and observed values. Additionally, goodness of fit was determined by a Bangdiwala’s agreement plot. Perfect matches were weighted as 1 (represented as black squares), and adjacent categories were weighted as 0.5 (grey squares) and lack of match between predicted and observed values were represented as white squares in the estimation of the Bangdiwala’s statistic. Differences between metastatic and non-metastatic patients and between tumor types (papillary *vs.* *in situ*) were assessed using the Wilcoxon-Mann-Whitney test. *P*-values < 0.05 were considered statistically significant. All statistical analyses were performed using R (version 4.0.2) and R packages clickR (version 0.4.47), glmnetcr (version 1.06), ordinal (version 2019.12-10) and vcd (version 1.4-7).

**REFERENCES**

1. Ramón-Núñez LA, Martos L, Fernández-Pardo Á, Oto J, Medina P, España F, et al. Comparison of protocols and RNA carriers for plasma miRNA isolation. Unraveling RNA carrier influence on miRNA isolation. PLoS One*.* 2017;12:e0187005.

2. Oto J, Navarro S, Larsen AC, Solmoirago MJ, Plana E, Hervás D, et al. MicroRNAs and Neutrophil Activation Markers Predict Venous Thrombosis in Pancreatic Ductal Adenocarcinoma and Distal Extrahepatic Cholangiocarcinoma. Int J Mol Sci*.* 2020;21:840.

3. Oto J, Plana E, Fernández-Pardo Á, Cana F, Martínez-Sarmiento M, Vera-Donoso CD, et al. Identification of mir-29c-3p as a robust normalizer for urine microrna studies in bladder cancer. Biomedicines*.* 2020;8:1–15.

4. Oto J, Plana E, Solmoirago MJ, Fernández-Pardo Á, Hervás D, Cana F, et al. microRNAs and Markers of Neutrophil Activation as Predictors of Early Incidental Post-Surgical Pulmonary Embolism in Patients with Intracranial Tumors. Cancers (Basel)*.* 2020;12:1536.

5. Oto J, Herranz R, Plana E, Sánchez-González JV, Pérez-Ardavín J, Hervás D, et al. Identification of miR-20a-5p as Robust Normalizer for Urine microRNA Studies in Renal Cell Carcinoma and a Profile of Dysregulated microRNAs. Int J Mol Sci*.* 2021;22:7913.

6. Xie F, Wang J, Zhang B. RefFinder: a web-based tool for comprehensively analyzing and identifying reference genes. Funct Integr Genomics*.* 2023;23:125.

7. Dweep H, Gretz N, Sticht C. miRWalk Database for miRNA–Target Interactions. 2014; p. 289–305.

8. Sticht C, De La Torre C, Parveen A, Gretz N. miRWalk: An online resource for prediction of microRNA binding sites. PLoS One*.* 2018;13:e0206239.

9. Van Nostrand EL, Pratt GA, Shishkin AA, Gelboin-Burkhart C, Fang MY, Sundararaman B, et al. Robust transcriptome-wide discovery of RNA-binding protein binding sites with enhanced CLIP (eCLIP). Nat Methods*.* 2016;13:508–14.

10. Lorenz DA, Her HL, Shen KA, Rothamel K, Hutt KR, Nojadera AC, et al. Multiplexed transcriptome discovery of RNA-binding protein binding sites by antibody-barcode eCLIP. Nat Methods*.* 2023;20:65–9.

**SUPPLEMENTARY TABLES AND FIGURES**

**Table S1. Clinical characteristics of the BC patients and healthy controls studied.**

|  | | **BC patients** | | **Controls** | | |
| --- | --- | --- | --- | --- | --- | --- |
|  | | **Screening (n=35)** | **Validation (n=172)** | **Screening (n=15)** | **Validation (n=94)** |  |
| **Age, *y*** | | 67 (61-74) | 68.5 (63-74) | 64 (51-76) | 63.5 (56-68) |  |
| **Male sex, *N (%)*** | | 32 (91) | 145 (84) | 12 (80) | 80 (85) |  |
| **Urine creatinine, *mg/dL*** | | 76.5  (37.4-123.3) | 77.1  (53.1, 118.7) | 78.5  (49.6-100.2) | 110.9  (70.6, 160.0) |  |
| **Tumor Stage and Grade, *N (%)***  **TaG1**  **TaG3**  **T1G3**  **TaG2**  **T1G2**  **T2G2/T2G3/T3G3** | | 10 (29)  8 (23)  5 (14)  0 (0)  0 (0)  12 (34) | 33 (19)  14 (8)  30 (17)  70 (41)  8 (5)  17 (10) | -  -  -  -  -  - | -  -  -  -  -  - |  |
| **Histology**  **Urothelial-papillary**  **Urothelial-*in situ*** | | 35 (100)  0 (0) | 158 (92)  14 (8) | -  - | -  - |  |
| **Clinical follow-up, months** | | 27 (23-32) | 22 (15-32) | 21 (21-34) | 40 (21-46) |  |
| **Disease status at the end of the follow-up, *N (%)***  **Disease-free alive**  **Progression alive**  **Death by BC**  **Death by other** | | 20 (57)  4 (11)  8 (23)  3 (9) | 129 (75)  6 (4)  19 (11)  18 (10) | -  -  -  - | -  -  -  - |  |
| **Metastasis, *N (%)*** | 10 (29) | 15 (9) | - | - |  |  |
| **Sampling**  **At first BC diagnosis**  **At BC recurrence** | | 35 (100)  0 (0) | 89 (52)  83 (48) | -  - | -  - |  |

Continuous variables are presented as median and interquartile range. Categorical variables are presented as count and percentage. BC, bladder cancer.

**Table S2. Dysregulated miRNAs among the different subgroups of BC patients and controls identified in the screening stage with a univariate ordinal regression with FDR adjustment.**

| **miRNA** | **Selected for validation** | **Sequence** | **Mean Ct** | **OR** | **FDR corrected *p*-value** |
| --- | --- | --- | --- | --- | --- |
| miR-21-5p | yes | uagcuuaucagacugauguuga | 26.24 | 0.101 | 0.0002 |
| miR-93-5p | yes | caaagugcuguucgugcagguag | 29.97 | 0.334 | 0.0012 |
| miR-30a-5p | yes | uguaaacauccucgacuggaag | 28.34 | 3.213 | 0.0012 |
| miR-425-5p | yes | aaugacacgaucacucccguuga | 31.69 | 0.365 | 0.0012 |
| miR-99a-5p | yes | aacccguagauccgaucuugug | 29.58 | 3.360 | 0.0012 |
| miR-23a-3p | yes | gggguuccuggggaugggauuu | 28.41 | 0.242 | 0.0012 |
| miR-192-5p | yes | cugaccuaugaauugacagcc | 30.36 | 3.485 | 0.0012 |
| miR-191-5p | yes | caacggaaucccaaaagcagcug | 30.09 | 0.268 | 0.0012 |
| miR-215-5p | yes | augaccuaugaauugacagac | 30.94 | 3.500 | 0.0012 |
| miR-130b-3p | no | cagugcaaugaugaaagggcau | 35.06 | 0.499 | 0.0015 |
| miR-210-3p | no | cugugcgugugacagcggcuga | 31.08 | 0.413 | 0.0015 |
| miR-10b-5p | yes | uacccuguagaaccgaauuugug | 28.56 | 3.115 | 0.0015 |
| miR-194-5p | no | uguaacagcaacuccaugugga | 29.49 | 3.142 | 0.0016 |
| miR-324-5p | no | cgcauccccuagggcauuggug | 33.97 | 0.378 | 0.0023 |
| miR-25-3p | no | cauugcacuugucucggucuga | 29.98 | 0.541 | 0.0023 |
| miR-100-5p | no | aacccguagauccgaacuugug | 32.37 | 2.985 | 0.0023 |
| miR-362-3p | yes | aacacaccuauucaaggauuca | 34.30 | 3.021 | 0.0023 |
| miR-200c-3p | yes | uaauacugccggguaaugaugga | 27.10 | 0.322 | 0.0023 |
| miR-320d | no | aaaagcuggguugagagga | 33.23 | 0.373 | 0.0023 |
| miR-30e-3p | no | cuuucagucggauguuuacagc | 31.53 | 3.133 | 0.0023 |
| miR-125b-5p | no | ucccugagacccuaacuuguga | 29.77 | 2.639 | 0.0030 |
| miR-30c-5p | no | uguaaacauccuacacucucagc | 28.47 | 2.758 | 0.0045 |
| miR-15b-3p | no | cgaaucauuauuugcugcucua | 36.76 | 0.716 | 0.0049 |
| miR-425-3p | no | aucgggaaugucguguccgccc | 34.26 | 0.478 | 0.0052 |
| miR-146a-5p | no | ugagaacugaauuccauggguu | 33.21 | 0.538 | 0.0052 |
| miR-30b-5p | no | uguaaacauccuacacucagcu | 28.75 | 2.752 | 0.0053 |
| miR-106b-5p | no | uaaagugcugacagugcagau | 31.30 | 0.442 | 0.0057 |
| miR-19a-3p | no | ugugcaaaucuaugcaaaacuga | 28.53 | 0.455 | 0.0060 |
| let-7c-5p | no | ugagguaguagguuguaugguu | 29.96 | 3.621 | 0.0075 |
| miR-320c | no | aaaagcuggguugagagggu | 31.07 | 0.402 | 0.0085 |
| miR-205-5p | no | uccuucauuccaccggagucug | 29.11 | 0.617 | 0.0085 |
| miR-148b-3p | no | ucagugcaucacagaacuuugu | 33.16 | 0.382 | 0.0085 |
| miR-34a-5p | no | uggcagugucuuagcugguugu | 31.22 | 0.493 | 0.0085 |
| miR-423-3p | no | agcucggucugaggccccucagu | 31.31 | 0.343 | 0.0085 |
| miR-19b-3p | no | ugugcaaauccaugcaaaacuga | 28.25 | 0.444 | 0.0085 |
| miR-150-5p | no | ucucccaacccuuguaccagug | 34.56 | 0.693 | 0.0095 |
| miR-17-5p | no | caaagugcuuacagugcagguag | 30.80 | 0.448 | 0.0107 |
| miR-141-3p | no | uaacacugucugguaaagaugg | 28.27 | 0.358 | 0.0113 |
| miR-16-5p | no | uagcagcacguaaauauuggcg | 27.23 | 0.670 | 0.0116 |
| miR-24-3p | no | uggcucaguucagcaggaacag | 28.83 | 0.390 | 0.0116 |
| miR-106a-5p | no | aaaagugcuuacagugcagguag | 30.31 | 0.454 | 0.0137 |
| miR-151a-3p | no | cuagacugaagcuccuugagg | 32.65 | 0.504 | 0.0144 |
| miR-15b-5p | no | uagcagcacaucaugguuuaca | 31.06 | 0.609 | 0.0157 |
| miR-15a-5p | no | uagcagcacauaaugguuugug | 31.29 | 0.658 | 0.0182 |
| miR-484 | no | ucaggcucaguccccucccgau | 32.87 | 0.550 | 0.0182 |
| miR-20a-5p | no | uaaagugcuuauagugcagguag | 29.79 | 0.482 | 0.0182 |
| miR-320b | no | aaaagcuggguugagagggcaa | 31.34 | 0.469 | 0.0182 |
| miR-320a | no | aaaagcuggguugagagggcga | 29.68 | 0.442 | 0.0182 |
| miR-92a-3p | no | uauugcacuugucccggccugu | 29.17 | 0.547 | 0.0186 |
| miR-126-3p | no | ucguaccgugaguaauaaugcg | 33.33 | 0.801 | 0.0186 |
| miR-584-5p | no | uuaugguuugccugggacugag | 36.88 | 0.711 | 0.0189 |
| miR-197-3p | no | uucaccaccuucuccacccagc | 32.69 | 0.551 | 0.0190 |
| let-7i-5p | no | ugagguaguaguuugugcuguu | 30.26 | 0.509 | 0.0194 |
| miR-652-3p | no | aauggcgccacuaggguugug | 32.46 | 0.509 | 0.0208 |
| miR-375 | no | uuuguucguucggcucgcguga | 32.11 | 1.428 | 0.0248 |
| miR-18a-5p | no | uaaggugcaucuagugcagauag | 35.74 | 0.835 | 0.0289 |
| miR-223-3p | no | ugucaguuugucaaauacccca | 27.84 | 0.735 | 0.0320 |
| miR-142-3p | no | uguaguguuuccuacuuuaugga | 31.02 | 0.753 | 0.0320 |
| miR-483-5p | no | aagacgggaggaaagaagggag | 37.56 | 0.854 | 0.0320 |
| miR-423-5p | no | ugaggggcagagagcgagacuuu | 32.40 | 0.561 | 0.0342 |
| miR-660-5p | no | uacccauugcauaucggaguug | 31.74 | 2.168 | 0.0357 |
| miR-451a | no | aaaccguuaccauuacugaguu | 28.41 | 0.843 | 0.0357 |
| miR-30d-5p | no | uguaaacauccccgacuggaag | 28.74 | 2.402 | 0.0376 |
| miR-186-5p | no | caaagaauucuccuuuugggcu | 33.89 | 0.542 | 0.0376 |
| miR-877-5p | no | guagaggagauggcgcaggg | 36.05 | 0.753 | 0.0377 |
| miR-2110 | no | uuggggaaacggccgcugagug | 31.06 | 1.473 | 0.0382 |
| miR-143-3p | no | ugagaugaagcacuguagcuc | 35.26 | 0.756 | 0.0431 |
| miR-27a-3p | no | uucacaguggcuaaguuccgc | 29.35 | 0.464 | 0.0475 |
| miR-185-5p | no | uggagagaaaggcaguuccuga | 32.31 | 0.745 | 0.0475 |
| let-7b-3p | no | cuauacaaccuacugccuuccc | 33.24 | 1.579 | 0.0487 |

OR: odss ratio; FDR: false discovery rate.

**Table S3: Significance level from the ANOVA test comparing pairwise the expression level of the dysregulated miRNAs in the screening cohort.**

| **miRNA** | **Control *vs.* TaG1** | **Control *vs.* TaT1G3** | **Control *vs.* T≥2GX** | **TaG1 vs. TaT1G3** | **TaG1 *vs.* T≥2GX** | **TaT1G3 *vs.* T≥2GX** |
| --- | --- | --- | --- | --- | --- | --- |
| miR-93-5p | 0.079 | **0.004** | **<0.001** | 0.836 | 0.092 | 0.343 |
| miR-362-3p | 0.848 | **<0.001** | **0.003** | **0.007** | 0.055 | 0.870 |
| miR-191-5p | **0.036** | **0.001** | **<0.001** | 0.762 | 0.449 | 0.940 |
| miR-200c-3p | >0.999 | **0.004** | **0.002** | **0.010** | **0.006** | 0.994 |
| miR-192-5p | 0.938 | **0.031** | **<0.001** | 0.198 | **0.003** | 0.252 |
| miR-21-5p | 0.436 | **0.037** | **<0.001** | 0.724 | **<0.001** | **<0.001** |
| miR-221-3p | 0.583 | **0.024** | 0.618 | 0.491 | 0.999 | 0.378 |

Significant *p*-values are depicted in bold.

**Table S4**: **Significance level from the ANOVA test comparing pairwise the expression level of the dysregulated miRNAs in the validation cohort.**

| **miRNA** | **Control *vs.* TaG1** | **Control *vs.* TaT1G2** | **Control *vs.* TaT1G3** | **Control *vs.* T≥2GX** | **TaG1 *vs.* TaT1G2** | **TaG1 *vs.* TaT1G3** | **TaG1 *vs.* T≥2GX** | **TaT1G2 *vs.* TaT1G3** | **TaT1G2 *vs.* T≥2GX** | **TaT1G3 *vs.* T≥2GX** |
| --- | --- | --- | --- | --- | --- | --- | --- | --- | --- | --- |
| miR-93-5p | 0.571 | 0.141 | **<0.001** | **<0.001** | 0.999 | **<0.001** | **0.007** | **<0.001** | **0.004** | 0.831 |
| miR-362-3p | 0.323 | **0.001** | **<0.001** | **0.004** | 0.767 | 0.442 | 0.350 | 0.938 | 0.780 | 0.980 |
| miR-191-5p | 0.514 | 0.659 | **<0.001** | **0.007** | 0.983 | **0.012** | 0.297 | **<0.001** | 0.076 | 0.979 |
| miR-200c-3p | 0.285 | **<0.001** | **<0.001** | 0.227 | 0.794 | **<0.001** | 0.986 | **<0.001** | 0.998 | **0.017** |
| miR-192-5p | 0.759 | 0.406 | 0.420 | 0.054 | 0.106 | 0.115 | **0.013** | 0.999 | 0.452 | 0.626 |
| miR-21-5p | 0.541 | **0.048** | **<0.001** | **<0.001** | 0.983 | **<0.001** | **<0.001** | **<0.001** | **<0.001** | 0.980 |
| miR-221-3p | 0.977 | >0.999 | 0.537 | **0.001** | 0.972 | 0.955 | **0.018** | 0.537 | **0.001** | 0.061 |
| miR-30a-5p | 0.998 | **0.043** | **0.012** | **0.013** | 0.124 | **0.037** | **0.022** | 0.910 | 0.533 | 0.904 |
| miR-425-5p | 0.432 | **<0.001** | **<0.001** | **<0.001** | 0.576 | **<0.001** | **0.002** | **<0.001** | **0.024** | 0.560 |
| miR-99a-5p | >0.999 | 0.114 | **<0.001** | **<0.001** | 0.428 | **0.008** | **<0.001** | 0.182 | **0.008** | 0.441 |
| miR-23a-3p | 0.314 | 0.269 | **<0.001** | **<0.001** | 0.995 | 0.127 | **0.038** | **0.009** | **0.005** | 0.823 |
| miR-215-5p | 0.917 | 0.292 | 0.215 | **0.013** | 0.160 | 0.112 | **0.007** | 0.990 | 0.248 | 0.494 |
| miR-10b-5p | 0.996 | **0.010** | **<0.001** | **<0.001** | 0.235 | **0.018** | **<0.001** | 0.558 | **0.021** | 0.337 |

Significant *p*-values are depicted in bold.

**Table S5. Validated and predicted targets of the 13 dysregulated miRNAs in BC.** These target proteins were identified using miRWalk 2.0 and were further integrated within the *bladder cancer* pathway. Validated targets are defined as those that have been empirically proven to be regulated by a miRNA. Predicted targets are defined as those that have been theoretically estimated based on the free binding energy between the miRNA and the presumed target mRNA sequence. miRNA targets validated with the miR-eCLIP assay are depicted in bold.

|  | ***Bladder cancer* pathway** | |
| --- | --- | --- |
| **miRNA** | **Validated targets** | **Predicted targets** |
| miR-221-3p | *E2F3,* ***MDM2****, RB1, TP53* | ***THBS1****, EGF, FIGF* |
| miR-93-5p | ***CCND1****, CDKN1A, DAPK3, E2F1, E2F2, E2F3, IL-8,* ***MAPK1****, MDM2, MYC, RB1, RPS6KA5, VEGFA* | *EGFR, DAPK2, EGF, MMP2, MAPK3, THBS1, VEGFB, EGF, ERBB2, MAP2K2, RASSF1, TP53, CDH1, FIGF* |
| miR-362-3p | *E2F1, VEGFA* | *DAPK1* |
| miR-191-5p | *BRAF* | *DAPK1, E2F3* |
| miR-200c-3p* | *E2F3, KRAS, VEGFA* | *MYC* |
| miR-192-5p* | *CDKN2A, RB1* | *CDK4, RASSF1, DAPK2* |
| miR-21-5p | *E2F1, E2F2,* ***E2F3****, EGFR, ERBB2, MMP2, MMP9, MYC, RB1, VEGFA* | *-* |
| miR-30a-5p | *CDH1, EGFR, MAPK1, THBS1, TP53* | *RPS6KA5* |
| miR-425-5p | *CCND1, E2F3, FGFR3, MDM2, NRAS* | *FIGF* |
| miR-99a-5p | *FGFR3, RB1* | *-* |
| miR-23a-3p | *CDH1, IL-8, MYC* | *E2F2,* ***CCND1****, RPS6KA5* |
| miR-215-5p | *CDKN2A, RB1* | *-* |
| miR-10b-5p | *CDKN1A, CDKN2A, TP53* | *RAF1* |

*The expression of these miRNAs was so low in the cells that no chimeric reads were found. Only 6 of the 76 computationally predicted targets (8%) were actually bound to the miRNA in the cells; however, many more not predicted were verified as targets with the miR-eCLIP. Among the most regulated, *CCND1* was the main target of miR-93-5p and miR-245-5p, regulation that was also computationally predicted.

**
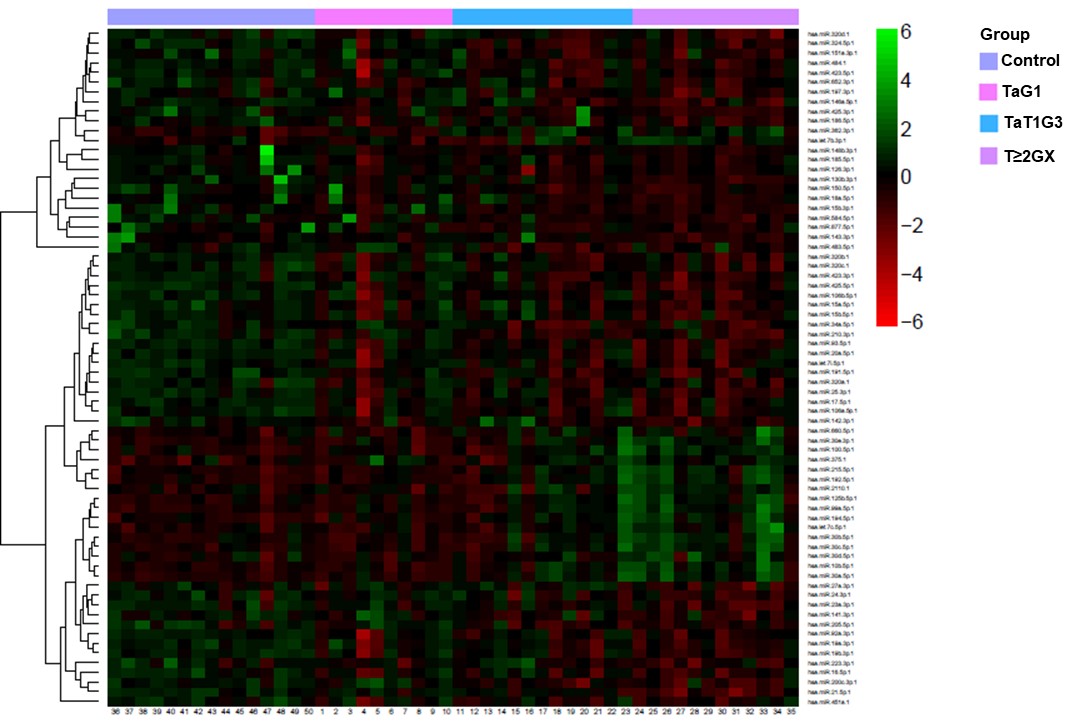
**

**Figure S1.** **Heatmap of the 70 dysregulated miRNAs according to the univariate ordinal regression approach with FDR adjustment comparing the different BC groups and healthy controls of the screening cohort**. Green represents miRNA underexpression and red represents overexpression.

**
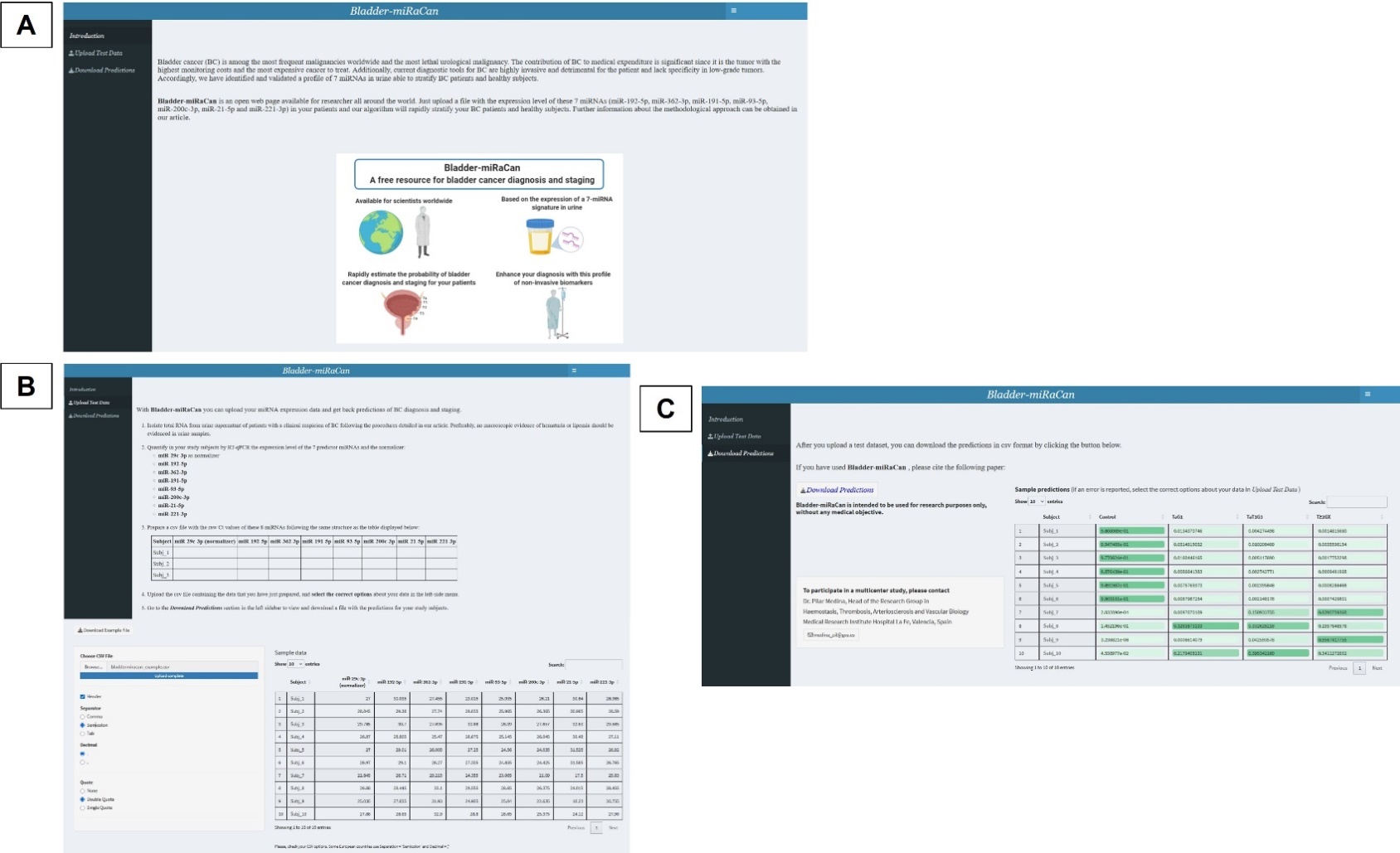
**

**Figure S2. Interface of the BladdermiRaCan app (**[**https://remote.iislafe.san.gva.es/sample-apps/bladder_miracan/**](https://remote.iislafe.san.gva.es/sample-apps/bladder_miracan/)**) to easily predict BC diagnosis and stage using our model.** This user-friendly interface empowers researchers to effortlessly predict the diagnosis and stage of their BC patients. Researchers can seamlessly upload the raw Ct values of the seven miRNAs and the normalizer via a straightforward interface. Subsequently, the application generates a downloadable file containing the predictions of belonging to the distinct analyzed categories for each studied subject. **A** Introduction explaining the applicability of the website; **B** submenu of data upload as raw Ct values of the seven miRNAs and the normalizer, to calculate the probability of belonging to the different categories analyzed for each subject studied. To facilitate the use of the app, a downloadable example file has been added to try an example; **C** submenu to download the file encompassing the predictions of belonging to the distinct analyzed categories (different BC stages-grades or healthy controls), where the present manuscript will be added for further information.

**
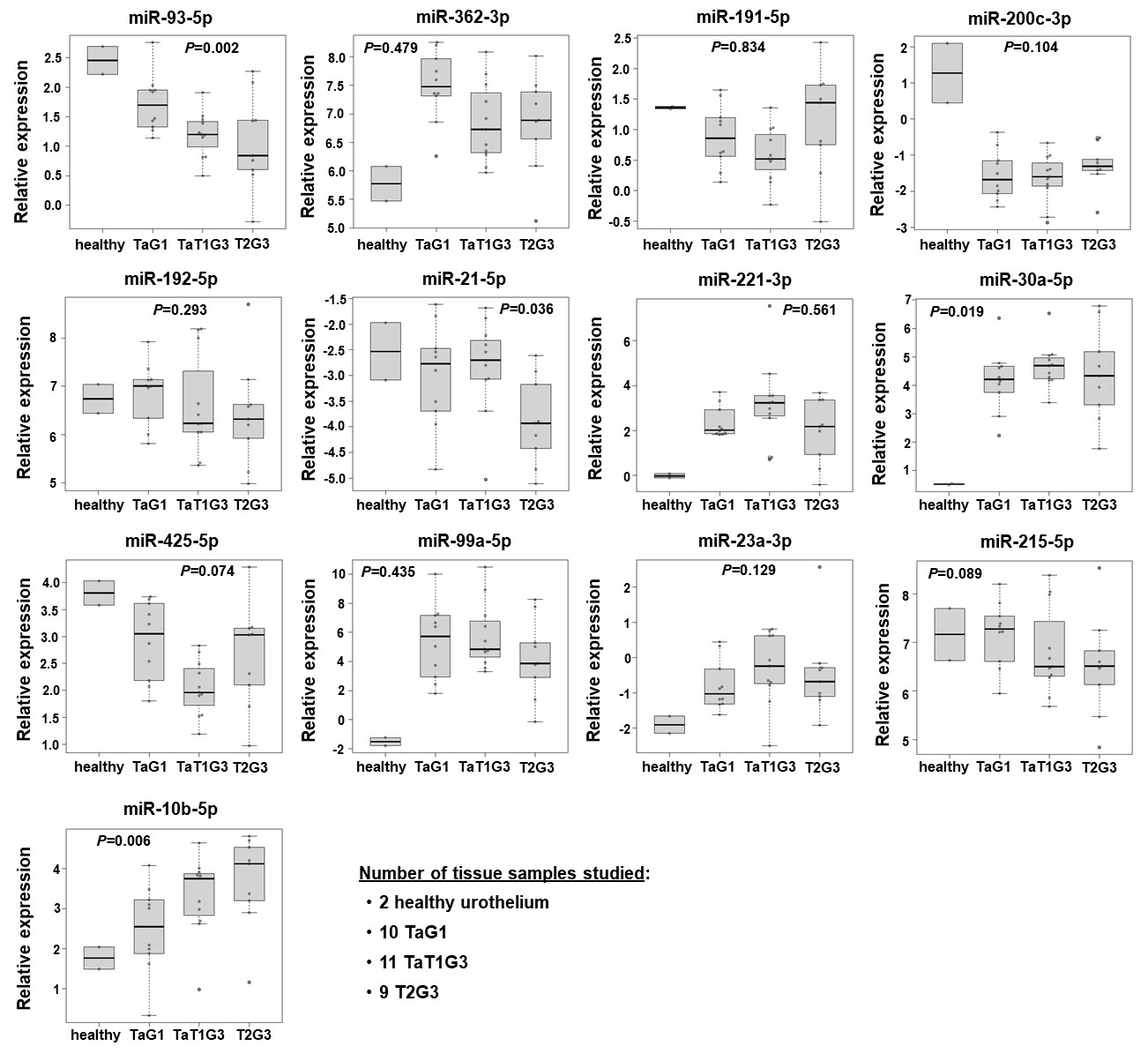
**

**Figure S3**. **Relative expression in FFPE tissue sections of the dysregulated miRNAs found in urine of the same BC patients**. All the 13 dysregulated miRNAs had an optimal expression value in tissue sections (Ct <35). The differences and trends in expression levels in tissue could be verified using univariate ordinal regression models. The results obtained in tissue are in the same direction to those obtained in urine, being the following miRNAs significantly dysregulated in tissue: miR-93-5p (Odds ratio, OR=0.114; 95% Confidence Interval, CI [0.026, 0.400]; p=0.002), miR-21-5p (0.474; [0.225, 0.929]; 0.036), miR-30a-5p (1.922; [1.124, 3.443]; 0.019) and miR-10b-5p (2.546; [1.356, 5.231]; 0.006).

**
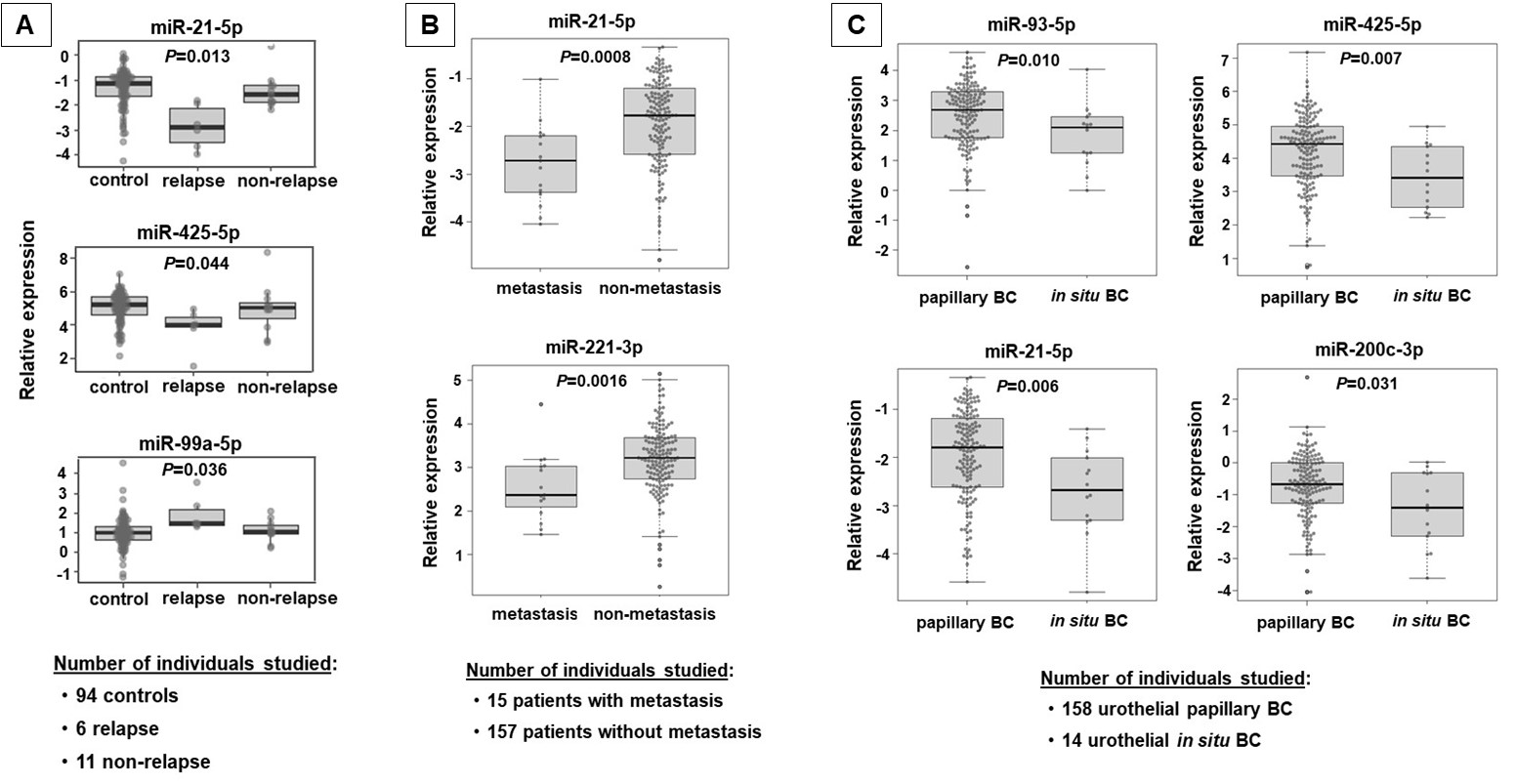
**

**Figure S4.** **Relative expression of dysregulated miRNAs according to tumor relapse, metastasis and BC cellular subtype** **in BC patients of the validation cohort. A** Relative expression of miR-21-5p, miR-425-5p and miR-99a-5p according to tumor relapse, measured in the sample right before relapse diagnosis and in the last sample collected from BC patients without recurrence, and in the only sample from controls. **B** Relative expression of miR-21-5p and miR-221-3p depending on the presence of tumor metastasis. **C** Relative expression of miR-93-5p, miR-425-5p, miR-21-5p and miR-200c-3p depending on the BC cellular subtype.
